# Accuracy of Drill Sleeve Housing in 3D-Printed and Milled Implant Surgical Guides: A 3D Analysis Considering Machine Type, Layer Thickness, Sleeve Position, and Steam Sterilization

**DOI:** 10.3390/bioengineering12080799

**Published:** 2025-07-25

**Authors:** Anna Seidel, Kai Zerrahn, Manfred Wichmann, Ragai Edward Matta

**Affiliations:** Department of Prosthodontics, University Hospital Erlangen, Friedrich-Alexander Universität Erlangen-Nürnberg (FAU), Glückstraße 11, 91054 Erlangen, Germany; anna.seidel@uk-erlangen.de (A.S.); kai.zerrahn@fau.de (K.Z.);

**Keywords:** static computer-aided implant surgery, surgical guide, drilling template, 3D printing, additive fabrication, CAD/CAM, implant accuracy, implantology, digital dentistry

## Abstract

Successful dental implant therapy relies on accurate planning and placement, e.g., through static, computer-aided implant surgery using CAD/CAM-fabricated surgical guides. This study examined production methods’ influence on surgical guide sleeve housing geometry. A model with two edentulous spaces was digitized using intraoral scanning and CBCT, and two virtually positioned implants were planned. Ten guides per group were produced using milling (MCX5), DLP printing (ASIGA and SHERA), and SLA printing (FORM), printing with 50 µm and 100 µm layers each. Each guide (n = 70) was then digitized using an industrial scanner before and after sterilization. Superimposition of the actual guide data with the reference data allowed for evaluation of deviations at the drill sleeve housing along the x-, y-, z-, and dxyz-axes. Descriptive and statistical evaluation was performed (significance level: *p* ≤ 0.0125). Significant differences existed among the production methods: Milling and SLA showed higher deviations than the DLP group (*p* < 0.001). Milled guides post-sterilization showed the highest deviations (0.352 ± 0.08 mm), while one DLP printer at 50 μm layer thickness showed lowest deviations (0.091 ± 0.04 mm). The layer thickness was insignificant, whereas sterilization increased deviation (*p* < 0.001). DLP produced the most precise implant surgical guides. All 3D printers were suitable for fabricating clinically acceptable surgical guides.

## 1. Introduction

Since Brånemark’s introduction of implantology in dentistry, implant-prosthetic treatment for edentulous patients has become a fundamental aspect of dental care [1]. The success of such treatments is contingent upon appropriate preparation of the implant site, ensuring primary stability, preventing infection of the bone bed, and avoiding premature loading in compromised bone conditions [2]. A critical factor is the precise three-dimensional placement of the implant in accordance with the prosthodontic plan [3,4]. There exist two principal methodologies: intraoperative planning, which relies on the existing tissue followed by subsequent prosthetic adaptation, and the contemporary approach of ‘backward planning’ [4]. In the latter, the desired prosthetic restoration is preoperatively planned, and the surgical procedure is virtually designed based on diagnostic data, including intraoral scans, three-dimensional imaging, and prosthetic models. This virtual implant positioning can subsequently be executed fully guided in static computer-aided implant surgery with the assistance of surgical guides [4,5]. Nonetheless, research has indicated that, despite meticulous planning and optimally conducted implant surgery, the implant positioning achieved in the patient frequently diverges from the virtual planning [6,7,8]. In addition to challenges encountered during the implantation procedure itself, which may arise from the application, technique, or the surgeon, another potential source of error in translating virtual plans to the patient is the surgical guide [2]. Following the virtual design of the surgical guide, it must be materialized. In the current era of digital dentistry and CAD/CAM technology, these surgical guides are typically manufactured using either subtractive or additive methods [9].

The development of digital technologies in this field began in the 1970s and has progressed continuously since then [10]. Subtractive manufacturing enables the realization of CAD-designed objects by milling or grinding preformed material blocks and discs [11]. In this process, material is removed from a blank using dry milling or wet grinding techniques based on digital data, executed via computer numeric controlled (CNC) machines. In dental practices, compact four-axis milling units are common, while dental laboratories typically use five-axis systems, allowing the production of larger and more complex structures [12]. Proper object orientation within the material block optimizes accuracy, efficiency, and material usage, though it may necessitate additional support structures. Despite producing more waste than additive manufacturing, subtractive methods often offer immediate usability and high material quality [13].

In additive manufacturing, objects are created layer by layer by thermally or chemically bonding material. The digital model is sliced along the build axis, and the geometry determines the build volume [14]. Various technologies are used for material deposition, including powder beds, extrusion, and light-based polymerization [15]. In dentistry, vat photopolymerization is widely applied for producing resin-based objects. A photosensitive liquid resin is selectively cured layer by layer using light. Common file formats like standard tessellation language (STL) are imported into slicer software, where parameters such as layer thickness, orientation, and support structures are defined [16]. Layer thickness can influence detail resolution and printing time, with typical dental values between 50 and 200 µm. Proper positioning on the build platform affects precision, material consumption, and waste. Manual post-processing includes support removal, surface refinement, and optional polishing, sterilization, or coating. The stereolithography (SLA) process utilizes a focused laser beam to selectively cure liquid resin within a single layer plane [17]. The laser’s small spot size (typically 60–120 µm) enables high-resolution fabrication [9]. The beam is directed along the x and y-axes using a pair of galvanometer-controlled mirrors, achieving precise positioning within the micrometre range. In contrast to SLA, digital light processing technology (DLP) cures resin by projecting an entire layer simultaneously using UV light and a digital micromirror device (DMD) [18]. The DMD contains independently controllable micromirrors that reflect light through a lens system and the transparent bottom of the resin tank onto the build platform. The resolution in the xy-plane is determined by the number and size of these micromirrors and the optical setup, typically achieving around 50 µm [14,19]. Because the full layer is exposed in a single projection, DLP enables faster print times than SLA [20,21].

The insertion of dental implants into the alveolar bone is conducted under sterile conditions. Steam sterilization is an effective and widely used method for the sterilization of medical devices, including 3D-printed surgical guides [22]. However, the thermal and mechanical stresses imposed by steam sterilization can alter the mechanical, chemical, and dimensional properties of these polymer-based devices [23]. These changes are primarily attributed to additional post-curing of residual monomers and the evaporation of absorbed water within the resin matrix [24,25].

Inadequate support of the surgical guide, imprecise fit, or inaccurate production might significantly affect clinical outcomes [26]. The drill sleeve in particular, as an integral component of the surgical guide, is crucial in fully guided implant placement: it ensures that the drill is accurately guided during osteotomy, thereby controlling the implant position across all three planes.

In the domain of digital planning, the alignment of planned implants corresponds to that of the associated sleeves and the cylindrical housings within the surgical guide where these sleeves are positioned and secured. Consequently, the three-dimensional orientation in the x-, y-, and z-axes of such sleeve housings can be regarded as a proxy for the implant’s axis. Ideally, in a surgical guide that is manufactured to precisely align with its CAD model, the axis of these cylindrical housings should coincide with that of the CAD model to clinically achieve the planned implant position, angulation, and depth.

The literature has yet to sufficiently explore the impact of modern manufacturing techniques on the accuracy of implant surgical guides, particularly concerning the precision of the sleeve housing, where deviations are presumed to have direct clinical implications on implant position. The aim of this study was to investigate the influence of the surgical guide production process (3D printing with different techniques and milling), the 3D printing layer thickness, the sterilization process, and the sleeve position on the accuracy of implant surgical guides regarding the x-, y-, z-, and dxyz-axes of the guides’ drill sleeve housings.

## 2. Materials and Methods

To investigate the deviation of the actual three-dimensional accuracy of the drill sleeve housing geometry in surgical guides, the following hypotheses were proposed for this study to elucidate the influencing factors:

1. There is a statistically significant difference in the three-dimensional deviation of the drill sleeve housing position of surgical guides between different machine types.
2. The layer thickness used by additive manufacturing machines (50 µm and 100 µm) has a statistically significant influence on the three-dimensional deviation of the drill sleeve housing position.
3. The position within the surgical guide (regions 11 and 14) has no statistically significant influence on the three-dimensional deviation of the drill sleeve housing position before and after sterilization.
4. Steam sterilization has a statistically significant influence on the three-dimensional deviation of the drill sleeve housing position.

**Figure 1:**
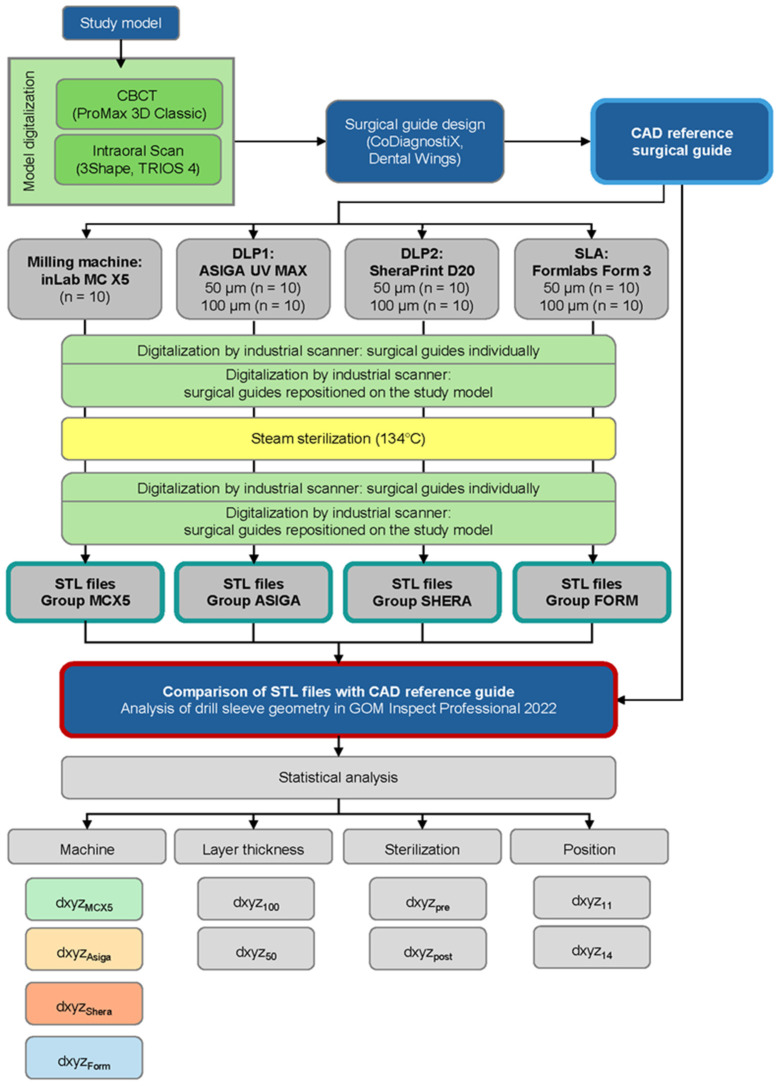
Flowchart of the study design and procedures with the individual groups under investigation.

The study encompassed the digitization of a study model, implant planning, design of a custom implant surgical guide, production through various methodologies, digitization of the templates, data analysis, and subsequent parametric and statistical analysis (Figure 1). The investigation encompassed three 3D printers and one 5-axis milling machine. Ten drilling templates were manufactured for each additive machine type at both 50 µm and 100 µm layer thicknesses (additive n = 60). Ten additional templates were created using the milling machine (subtractive n = 10), resulting in a total of 70 drilling templates for investigation. All objects under investigation were fabricated according to the manufacturer’s recommendations for surgical guides under expert supervision of dental technicians.

### 2.1. Design of Surgical Reference Guide

**Figure 2:**
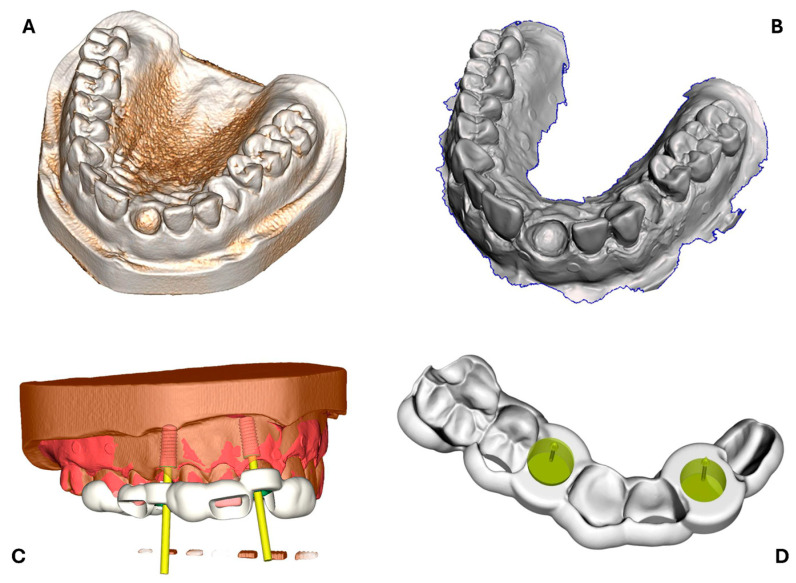
(**A**) Three-dimensional model of the CBCT scan; (**B**) intraoral scan with missing teeth in regions 11 and 14; (**C**) view of the surgical guide (white) and virtually positioned implants (red with implant axes in yellow) based on intraoral scan (red) and CBCT (brown) in the implant planning software; (**D**) reference surgical guide with drill sleeve housing geometry (green) at positions 11 and 14.

A study model made from radiopaque resin with edentulous spaces at positions 11 and 14 was digitized by intraoral scanning (Trios 4, 3Shape, Copenhagen, Denmark) and CBCT (Planmeca ProMax 3D Classic, Planmeca, Helsinki, Finland). Both the intraoral scan and the DICOM data of the CBCT scan were imported and superimposed in dedicated implant planning software (CoDiagnostiX, DentalWings GmbH, Chemnitz, Germany). Implant planning was performed at the edentulous spaces with two 10 × 4.1 mm bone-level implants (Straumann Bone Level Roxolid, Straumann AG, Basel, Switzerland). A drill guide for fully navigated implant placement was designed with an inner drill sleeve housing geometry of 5 mm diameter, 5 mm height, contact surfaces on teeth 16–21, 0.2 mm offset, a wall thickness of 3 mm, and two 10 mm windows proximal to teeth 12 and 15 to verify guide position. The data were exported in STL file format and served as the surgical CAD reference guide (Figure 2).

### 2.2. Surgical Guide Production

Comprehensive descriptions of technique, machine, materials, and post-processing measures for each method are provided in Table 1. Standardized protocols as recommended by the machine manufacturers were meticulously followed to minimize errors and ensure reproducibility and comparability of results.

Prior to the fabrication of each batch, both the 5-axis milling machine and all 3D printers used in this study were calibrated following the specific protocols provided by their respective manufacturers to ensure optimal accuracy and repeatability. The study investigated the production of surgical guides using one milling system and four different 3D printing devices, each representing a distinct manufacturing approach.

For subtractive manufacturing, the inLab MC X5 (Dentsply Sirona, Bensheim, Germany) was used and assigned to the MCX5 group. Additive manufacturing was performed using three different resin-based 3D printers: the Sheraprint D20 (Shera, Lemförde, Germany) and the Asiga Max UV (Asiga, Sydney, Australia), both operating with DLP technology and categorized into the SHERA and ASIGA groups, respectively, and the Form 3 (Formlabs Inc., Somerville, MA, USA), utilizing SLA technology, which formed the FORM group.

**Figure 3:**
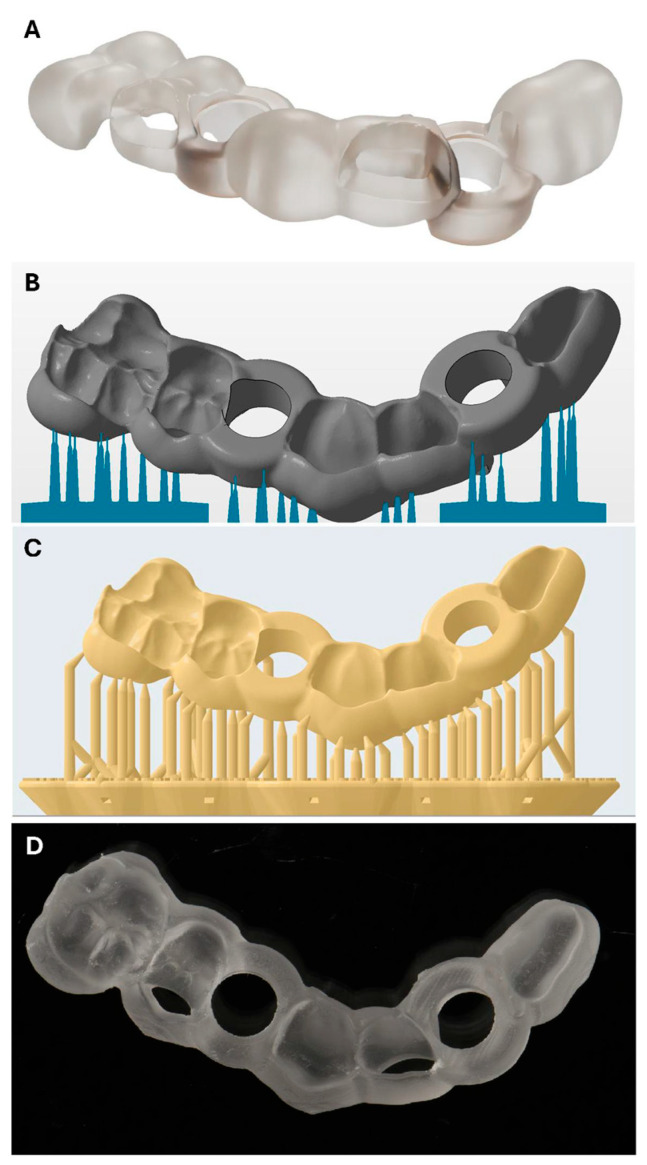
(**A**) CAD reference file of designed surgical guide; (**B**) surgical guide (grey) and support structures (blue) in the NetFabb 2020 Premium Slicer software with the applied 45° building angle; (**C**) surgical guide with support structures (yellow) in the PreForm Slicer software (Version 3.17) with the applied 45° building angle; (**D**) 3D-printed template of the Form group.

The STL file of the surgical guide was imported into the machine-specific nesting software. For all additive manufacturing groups, surgical guides were oriented at a 45° angle along the oro-vestibular axis. Support structures were automatically generated by the respective nesting software. Manual inspection and correction of the support structures were performed to ensure that sufficient support was present in critical areas, such as overhangs, and that no support structures were placed within the drill sleeve housing. All production settings were applied according to the manufacturers’ recommendations to ensure standardization and reproducibility (Figure 3).

For each production modality and, where applicable, each layer thickness, a total of ten surgical guides were fabricated, resulting in 60 additively manufactured (n = 10 per printer and layer thickness at 50 µm and 100 µm) and 10 milled specimens. All specimens were produced in accordance with the manufacturers’ specifications for surgical guides and under the expert supervision of dental technicians.

After production, all 3D-printed guides underwent standardized post-processing and post-curing procedures, in line with manufacturer specifications. Particular attention was given to the careful removal of support structures to avoid any alteration or damage to the surface of the surgical guides.

### 2.3. Digitalization and 3D Evaluation of Surgical Guides

#### 2.3.1. Surgical Guide Digitalization

Following production, the surgical guides were digitized both individually and in their final position on the study model using a high-precision industrial scanner.

To facilitate a comprehensive analysis of the drill sleeve housing axes and geometry, the surgical guides were digitized using a triple scan protocol, initially on the study model and subsequently individually, employing the ATOS SO II industrial scanner (GOM GmbH, Braunschweig, Germany): the surgical guides were individually positioned on the study model, and the guide was fixed to the model with adhesive wax. Reference points with a diameter of 0.8 mm (GOM GmbH, Braunschweig, Germany) were placed on the study model, and a thin coating of titanium oxide (Rutile Powder, GOM GmbH, Braunschweig, Germany) in an alcohol suspension was applied to all surgical guides and the study model using an airbrush to enhance scanner reproducibility. These reference points enabled the metrology software to automatically align and merge the individual scan data into a global coordinate system. By serving as fixed spatial anchors, they ensured consistent orientation of the subsequent object scans in the software. The resulting point clouds were then used to reconstruct high-resolution surface models for subsequent geometric analysis. The templates were subsequently digitized on the study model with an accuracy of approximately 4 µm using the industrial scanner [27]. Digitizing the surgical guide solely on the study model would have resulted in significant portions of the guide remaining obscured, particularly concerning the drill sleeve housing fit. To address this limitation, the triple scan protocol by Holst et al. [28] was employed to conduct a thorough examination of the surgical guides’ inner drill sleeve axes and geometry. Consequently, each guide was scanned individually at 360° within a metal frame with 0.4 mm diameter reference points (GOM GmbH, Braunschweig, Germany). Subsequently, the scans underwent polygonization to convert the generated point clouds into parametrically analysable CAD files. The resulting surface scans were then evaluated and referred to as ‘pre-sterilization’.

#### 2.3.2. Sterilization Process

After the first digitization, each surgical guide was cleaned thoroughly, packed in a single container, labelled, and sent in the central sterilization facility at the University Hospital Erlangen-Nuremberg. They were steam-sterilized at 134 °C in accordance with the guidelines established by the Robert Koch Institute [22].

After that, the surgical guides underwent another digitization round post-sterilization to assess the impact on accuracy as described in the previous section. The resulting surface scans were then evaluated and referred to as ‘post-sterilization’.

#### 2.3.3. Three-Dimensional Data Analysis and Calculation of Deviations

To assess inaccuracies in the production of the surgical guides, the scans of milled/printed guides were compared with their reference CAD guides using metrology software (GOM Inspect Suite; GOM GmbH). The collected data were compared with the CAD reference guide to calculate spatial deviations of the drill sleeve housings. The system measured both linear and angular deviations between the planned and actual sleeve housing axes. Digital evaluations were conducted at both drill sleeves, regions 11 and 14, to assess linear deviations of implant axes at the sleeve housing, as described by Matta et al. and Russo et al. [29,30].

**Figure 4:**
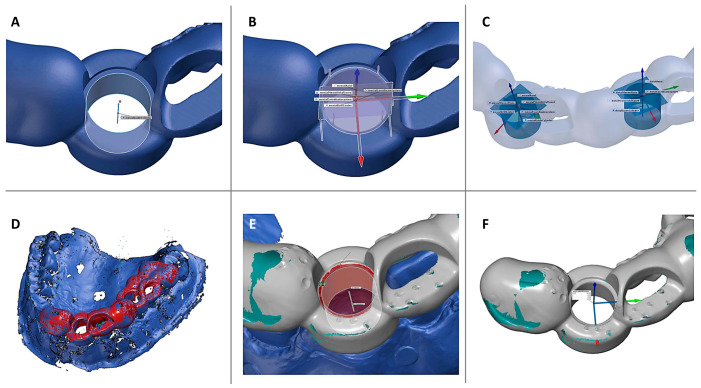
(**A**) The intersection point between the cylinder centreline and the support plane is established at the CAD reference guide (blue). (**B**) A local coordinate system is created at the intersection point between the planes perpendicular to the cylinder and at the top plateau of the drill sleeve. The x-axis (red) aligns oro-vestibularly, the y-axis (green) mesio-distally, and the z-axis (blue) apical-coronally. (**C**) An overview shows the designed geometries for positions 11 and 14, relating to drill sleeve housing in the surgical guide. (**D**) The data of the surgical guide scan (grey) are superimposed on the study model scan with repositioned guide (blue), previously superimposed on CAD reference. (**E**) The cylinder is constructed fitting the guide sleeve housing on the scanned printed guide (grey). (**F**) Results of the linear deviation of the drill sleeve housing of the actual surgery. The coordinate system of the CAD reference shown represents the reference point of the deviation measurement.

Initially, reference geometries were constructed in the CAD reference guide data: a plane at the sleeve top, followed by a cylinder fitted to the sleeve housing geometry, with a perpendicular plane (Figure 4). Measurement points for each drill sleeve were determined by the intersection of the cylinder axis with the vertical plane. The axes of the sleeve housings were calculated, which correspond to the planned implant axis. For the subsequent comparison of the actual surgical guides with the CAD reference, an independent local three-dimensional coordinate system was established based on the CAD reference guide. The origin of this coordinate system was defined at the centre of the drill sleeve housing shoulder. The coordinate axes were oriented as follows: the x-axis in the oro-vestibular direction, the y-axis in the mesio-distal direction, and the z-axis aligned with central axis of the cylindrical sleeve housing (apical–coronal direction) corresponding to the planned implant axis. As a second step, the scan data of the study model with the seated surgical guide was superimposed with the CAD reference model first by manual alignment followed by the best-fit algorithm. Subsequently, the individual scan of the corresponding surgical guide was superimposed first by manual alignment followed by the best-fit algorithm with the scan data of the surgical guide seated on the study model, which was already aligned in the previous step. This ensured that the repositioned surgical guide on the study model could be compared to the CAD reference surgical guide in alignment with the triple-scan protocol. For comparison, corresponding measurement points were constructed in the same way as described above based on the superimposed surgical guide. Comparison was made by measuring the linear deviation between the drill sleeve shoulder centre of the CAD reference guide (origin of the local coordinate system) and the drill sleeve shoulder centre of the repositioned surgical guide. The linear deviation at the sleeve entrance and the Euclidean distance dxyz were calculated. Deviations from the reference model were presented as means with standard deviation, minimum, and maximum in millimetres.

### 2.4. Statistical Analysis

The influence of machine type on the deviation in the drill sleeve housing, both before and after sterilization, was investigated by aggregating measurements from positions 11 and 14, with layer thicknesses of 50 µm and 100 µm, for both additive and subtractive manufacturing groups (n = 140). JASP (Version 0.19.1, University of Amsterdam, Netherlands) was utilized for statistical analysis of individual groups. Normal distribution and variance homogeneity were assessed with a significance level of *p* ≤ 0.2. Because of multiple hypothesis testing a Bonferroni correction was applied to the initial alpha error level of 5% to adjust the alpha error level to *p* ≤ 0.0125 to mitigate alpha error accumulation. A prerequisite examination ensured the statistical model’s appropriateness for the data. As no significant influence of layer thickness on drill sleeve deviation was observed in preliminary testing, this parameter was not included as an independent factor in the final group comparisons. Aggregating data across layer thickness levels allowed for more robust statistical testing given the limited subgroup sizes. To evaluate differences in drill sleeve housing geometry deviation between machine types before and after sterilization, the non-parametric Kruskal–Wallis test was applied. The Mann–Whitney U test was performed to analyse the difference between tooth position and the layer thickness of the additive machine types regarding the deviation of the drill sleeves before and after sterilization. A *t*-test for dependent samples evaluated the impact of sterilization.

## 3. Results

The results for the deviation of the surgical guide sleeve axis and geometric accuracy are presented in Table 2. A total of 10 guides were analysed for the subtractive method, and 20 guides for each additive fabrication method, with evaluations conducted on two surgical guide drill sleeves per specimen. Prior to the sterilization process, the smallest deviations for dxyz were observed at a 50 μm layer thickness in the DLP printer group: SHERA exhibited a mean deviation of 0.091 ± 0.04 mm, followed by ASIGA with 0.195 ± 0.14 mm (Table 2). At a printing layer thickness of 100 μm, the DLP printers again demonstrated superior accuracy, with ASIGA at 0.129 ± 0.07 mm, followed by SHERA at 0.183 ± 0.07 mm. Among the 3D printers, FORM exhibited the highest deviation for both layer thicknesses investigated, with 0.290 ± 0.12 mm at 50 μm and 0.233 ± 0.09 mm at 100 μm. The subtractive fabrication technique produced the least accurate surgical guide sleeves, with deviations double those of the DLP printers for dxyz, showing a mean deviation of 0.352 ± 0.08 mm. After sterilization, the mean values for dxyz deviation increased across all groups and layer thicknesses. Regarding the x-, y-, and z-axes, the highest deviations were observed in the z-axis, except for ASIGA 100. In the ASIGA group, guides fabricated with a 100 μm layer thickness were more accurate compared to those with a 50 μm layer thickness, as some outliers were noted for the smaller layer thickness.

The deviation of the Euclidian distance was statistically analysed for machine type, layer thickness, sleeve position, and sterilization as described in Section 2.4 (*p* < 0.0125). Median values for individual groups, both pre- and post-sterilization are presented in Table 3. Significance values when comparing the geometric deviation of surgical guide sleeve geometries for different machine types before and after sterilization are detailed in Table 4.

**Figure 5:**
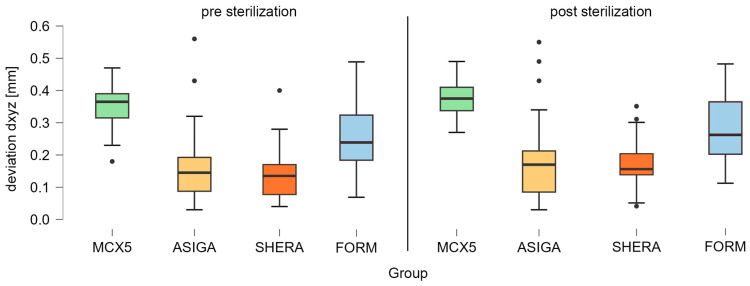
Boxplot of the Euclidean distance dxyz for 50 and 100 µm in total, measured at both drill sleeve positions 11 and 14 for the comparison before and after sterilization of the groups MCX5 (green), ASIGA (light orange), SHERA (dark orange), and FORM (blue). The outliers are represented as black dots.

No statistically significant differences were observed between the ASIGA and SHERA groups in any sterilization state, nor between the MCX5 and FORM groups prior to sterilization. However, all other comparisons demonstrated statistical significance. The type of machine significantly affected the accuracy of the surgical guide sleeve. Variations in layer thicknesses (50 and 100 µm) did not impact the deviation of the drill sleeve housings, either before or after sterilization. Another hypothesis posited that the position of the drill sleeve within the surgical guide (positions 11 and 14) would not influence the three-dimensional deviation of the drill collar of the template before and after sterilization. This hypothesis was confirmed, as statistical testing indicated that the position did not affect the accuracy of the drill sleeve housing, neither pre- nor post-sterilization. Regarding the influence of steam sterilization on the three-dimensional deviation of the drill sleeve geometry, the *t*-test conducted revealed a statistically significant difference between measurements taken before and after sterilization (*t*(139) = −4.741; *p* < 0.001; Figure 5). Thus, steam sterilization significantly impacted the deviation at the surgical guide drill sleeve housing.

## 4. Discussion

This study examined the impact of subtractive/additive manufacturing processes in surgical guides used for static computer-assisted implant surgery.

In particular, the layer thickness in additive manufacturing, the position of the drill sleeve within the surgical guide, and the effects of sterilization on the geometric deviation, particularly concerning the guides’ sleeve housing geometries in the x-, y-, and z-axes, were assessed by means of parametric 3D CAD analysis. These measurements, in conjunction with additional known data such as sleeve offset and planned implant length, enabled the estimation of linear errors in implant positioning at both the implant platform and the apex. While other sources of error may arise during implant placement, the findings of this study are specifically intended to analyse the potential outcomes when considering the deviation of the three-dimensional alignment of the sleeve housing from surgical guides in isolation.

When employing a novel manufacturing technique for dental appliances, the primary expectations that need to be achieved are accuracy and consistency. These attributes are crucial, particularly in the context of surgical guides, as they enable transferring the virtually planned implant position to the patient in static implant surgery. Upon evaluating the study’s results for these characteristics, the device that demonstrated the highest accuracy and consistency was found for the DLP printer group SHERA.

The present study identified significant differences in production methods both prior to and following sterilization. The findings indicated that the guides printed in the SHERA group exhibited the lowest deviation from the reference CAD guide, followed by ASIGA and FORM. Regarding manufacturing technologies, the most precise surgical guides were produced using DLP, followed by SLA, with the subtractive CAD/CAM fabrication method MCX5, a five-axis milling machine, yielding the least precision.

Other studies have examined the impact of manufacturing methods on implant position, surface deviation of the surgical guide, and deviation of the drill sleeve housings. Concerning the superiority of DLP over SLA found in this study, similar observations were reported by Keßler et al. and Gjelvold et al., who assessed the definitive implant position of implant surgical guides after production with different devices [31,32]. Regarding angular deviation, the printer from the SHERA group used in this study exhibited the smallest deviation of 0.76 ± 0.52°, and in contrast, a predecessor from the FORM group demonstrated a significantly higher deviation of up to 2.43 ± 0.64° when compared to the planned implant position during the realization of the implant position on the model [31]. Additionally, the linear horizontal displacement on the crestal ridge was notably higher with the SLA technology. This study concentrated on assessing the three-dimensional deviation of the drill sleeve housing geometry across all spatial planes, while it did not examine the deviation in implant angulation. Nevertheless, the findings of Gjelvold et al. are consistent with the results of this study, as they demonstrated that the guides produced by the DLP printer exhibited lower deviations, except for deviation in horizontal implant position [32].

Conversely, Anunmana et al. found no significant difference between DLP and SLA [33]. When examining the deviation of the surface in terms of trueness and precision of 3D-printed implant surgical guides, Wegmüller et al. and Morón-Conejo et al. observed a lower deviation for SLA compared to DLP technology by investigating the deviation of the surgical guides surface [34,35]. However, these authors quantitively analysed the deviation by superimposition of the single surgical guides with the reference data. Opposingly, in this study the surgical guide was compared to the reference data after reposition on the study model. Therefore, inaccuracies of the occlusal surface of the surgical guide might have been equilibrated in their analysis, whereas inaccuracies of the occlusal surface might have produced inadequate fitting on the study model. Similarly, Lo Russo et al. also reported a contrasting trend regarding the deviation of the drill sleeve housing, noting that milled surgical guides exhibited lower deviations compared to DLP printed guides: an additional lateral error of 0.2 mm would have to be anticipated at both the implant apex and the implant platform [36]. The authors superimposed the surgical guides with the reference data, rather than comparing them with the repositioned guide on the study model, which is more realistic to a clinical setting. This study employed a method for conducting three-dimensional measurements that is more clinically representative than merely aligning fabricated surgical guides with reference data. Specifically, each surgical guide was positioned on the study model prior to scanning, then scanned and thereafter matched with the reference guide data. While this approach enhances clinical realism, it inherently introduces user-dependent variability, which must be considered when interpreting the study results.

For a rigorous analysis of the findings, it is imperative to compare them with those reported in the existing literature. Nevertheless, it should be acknowledged that due to the heterogeneity of the study conditions—such as the methods of data analysis, evaluation parameters, and the devices and resins, which are continually being modified by the industry—an exact comparison of the results is not entirely feasible.

In addition to the inherent difficulty in comparing study results due to the heterogeneity of study designs, the appropriateness of comparing different printers, which belong to different price segments, is questionable. Depending on the resolution and technology, 3D printers for dental applications have a wide range in purchase and production costs. Consequently, when categorizing these results, it is important to consider that superior technology naturally incurs a higher cost. However, high precision is crucial to produce surgical guides for fully navigated implant surgery.

In the present study, significant differences were found regarding the influence of sterilization on the surgical guide sleeve housing accuracy. These findings align with observations made by Kessler et al. in an in vitro study [31]. They observed that sterilization significantly influenced the deviation of implant position after insertion compared to the planning. In contrast, a study by Marei et al. did not show a significant influence on the sterilization method on the accuracy of surgical guides produced by SLA [37]. Although the authors did reposition the surgical guide on the study model, the evaluation was performed by comparing sterilized vs. non-sterilized surgical guides, whereas in this study, all surgical guides in any sterilization state were compared to the original planning. While the findings are statistically significant, they do not hold clinical importance for minor deviations, such as 0.02 mm along the dxyz axis [18].

In contemporary implantology, efforts are directed towards planning implant placement in a prosthetically optimal position by selecting appropriate implant diameters and lengths. Drill guides are frequently used for flapless implant surgery or for implant placement according to virtually planned positions based on a prosthetic plan. Careful planning is required to ensure precise placement and predictable results. It can be clinically significant if the implant is positioned differently along its x-, y-, or z-axes than virtually planned. In this study, the most substantial deviations in the individual axes of the surgical guides were observed in the z-axis of the milling machine and 3D printer group FORM, with deviations from the planned guide averaging 0.287 ± 0.067 mm and 0.242 ± 0.122 mm, respectively. This deviation, amounting to an additional quarter of a millimetre in the z-axis, would result in the implants being placed at a shallower depth than originally intended. Clinical studies have confirmed this observation [38]. In a clinical context, this may necessitate manual depth correction, posing a risk of altering the drill orientation in other axes, thereby causing the implant position to deviate from the virtually planned position.

The layer thickness is an adjustable parameter that represents the build direction. The movement of the 3D printer’s building platform and the curing depth of the light source employed for polymerization may affect the accuracy in this plane. A study that examined the accuracy of 3D-printed dental implant models demonstrated higher deviations in the z-axis for the SLA printer compared to the two DLP printers that were investigated [39]. However, comparisons cannot be directly made as the surgical guides in this study were printed with a 45° build angle, obfuscating the direct impact of build direction on the deviation of drill guide sleeve housings.

Several factors have been identified as influencing the accuracy of 3D-printed restorations, templates, or models, which is essential for dental applications. In addition to the printing technology and layer thickness, which were examined in this study, other printing parameters, such as the build angle, placement on the build platform, and printing temperature, can also affect the quality of the products. Research has demonstrated that the build angle of an object within a 3D printer significantly affects the accuracy of the printed object [40]. Unkovskiy et al. identified that the most precise bar-like test specimen was achieved using an SLA printer with a 45° build angle [41]. Another investigation into the fit of 3D-printed prostheses for SLA printers revealed that the basal fit of restorations was inferior at 0° and 90° compared to 30° and 45° [42]. This phenomenon occurs because a shallow orientation (e.g., 0°) increases the printing area, thereby enhancing polymerization within layers rather than between them [43]. Insufficient interlayer polymerization can result in delamination, which is classified as a misprint. This effect was not observed in the current study. In accordance with the findings in the literature, a 45° build angle was adopted for our study and applied across all three printers analysed. The impact of the object’s orientation relative to the build plate was not examined in this study. The consistent application of a 45° build angle for all guides in the 3D printers utilized in this study suggests a standardized approach to this parameter. Future research could benefit from exploring the effects of varying build angles on the fabrication of drilling templates across different 3D printers.

After determining the building angle in the 3D printer software, support structures must be attached to drilling templates to ensure adhesion to the building platform. This process was automated and manually refined in the slicer software after visual inspection of vulnerable areas. Alharbi et al. demonstrated that thinner support structures yield higher surface accuracy compared to thicker ones [44]. However, this study did not evaluate the impact of varying support structures. The supports were adequately distributed, and no misprints occurred. The approach aligns with the settings of the dental lab or clinicians’ work.

The surgical guides were fabricated using different materials in each group (Table 1). Kessler et al. determined that the choice of material significantly influenced the accuracy of the implant surgical guide when compared to the planning with milling, DLP, and SLA [31]. Similarly, Sharma et al. observed the same effect when examining the influence of material selection on cubic test specimens [45]. It is important to note that in this study, the materials used and examined were those recommended by the printer manufacturer to produce surgical guides. These materials had an optimal polymerization wavelength of 385 nm for DLP and 405 nm for SLA, which was within the range of the printing system under investigation, and all were approved for sterilization. This approach was adopted to ensure that the results aligned with the user’s procedure and resulting outcome, as most users, laboratories, and dental technicians adhere to the specifications provided by the device manufacturer.

The methodology for evaluating the fit and accuracy of dental restorations and applications employed in this study is well established. Given the high precision of the industrial scanner utilized (ATOS, GOM GmbH), with an accuracy of less than 4 µm, it can be assumed that the digitization of the surgical guides is true to the original object [27]. Matta et al. also employed this industrial scanner to analyse both conventional and additively manufactured surgical guides [30]. They determined that the repeated repositioning of the surgical guide on the study model was highly reproducible.

## 5. Conclusions

Implantology constitutes a fundamental component of dental treatment. Surgical guides for static computer-aided implant surgery facilitate both partially and fully navigated implant placement. In this study, the accuracy of CAD/CAM fabricated surgical guides regarding the axial accuracy of the drill sleeve housings was evaluated through three-dimensional deviation assessments. The findings indicate significant differences between machine types before and after sterilization, with milled guides exhibiting greater deviations compared to 3D-printed ones. DLP-technique-based 3D printers produced more accurate guides with smaller deviations compared to the SLA printer. The applied layer thicknesses of 50 µm and 100 µm demonstrated no significant differences. Sterilization using moist heat resulted in significant deviations across all groups.

Overall, all investigated production methods showed clinically acceptable implant surgical guides. With the rapid development of additive technologies and fabrication methods in the industry, it is necessary to conduct ongoing research regarding their accuracy and clinical application.

## Figures and Tables

**Table 1:** Description of investigated groups, technology, machine, nesting software, resin materials, and post-processing devices.

Group	Technology	Machine	Nesting Software	Material	Post-Processing Units
MCX5	Milling	inLab MC X5	inLab CAM SW 2019 (Dentsply Sirona, Bensheim, Germany)	Splint Plus BioStar (ERNST HINRICHS DENTAL)	
FORM	SLA	Asiga Max UV	PreForm Version 3.17 (Formlabs Inc., Somerville, MA, USA)	Imprimo LC MJF (SCHEU-DENTAL)	Imprimo Clean and Imprimo Cure (SCHEU-DENTAL); nitrogen atmosphere
ASIGA	DLP	Shera D20	Asiga Composer Version 1.37 (Asiga, Sydney, Australia)	SHERAprint ortho plus UV (Shera Werkstoff-Technologie)	BioSonic UC150 (Coltene), Otoflash G171 (NK-Optik); nitrogen atmosphere
SHERA	DLP	Form 3	Netfabb Premium 2020 (Autodesk, San Francisco, CA, USA)	Surgical Guide Resin V1 (Formlabs)	Form Wash and Form Cure (Formlabs)

**Table 2:** Descriptive analysis of the analysed guides produced by the milling MCX5 (n = 10), DLP ASIGA (n = 20), DLP SHERA (n = 20), and SLA FORM (n = 20) groups: Euclidean distance dxyz (Mean), standard deviation (SD), minimum (Min), and maximum (Max) in mm and for the x-, y-, and z-axes as compared to the reference guide.

						Deviation [mm]					
				Pre-Sterilization				Post-Sterilization			
Machine	Evaluation Axis	Layer Thickness	Sleeve Position	Mean	SD	Min	Max	Mean	SD	Min	Max
MCX5	dxyz	-	total	0.352	±0.075	0.18	0.47	0.372	±0.055	0.27	0.49
	dxyz		11	0.307	±0.069	0.18	0.38	0.352	±0.045	0.27	0.42
	dxyz		14	0.396	±0.057	0.32	0.47	0.392	±0.062	0.28	0.49
	x		total	−0.149	±0.057	−0.26	−0.09	−0.173	±0.063	−0.29	−0.09
	y		total	0.108	±0.073	−0.04	0.25	0.111	±0.060	−0.02	0.24
	z		total	0.287	±0.067	0.13	0.40	0.297	±0.060	0.19	0.43
ASIGA	dxyz	50 µm	total	0.195	±0.136	0.04	0.56	0.205	±0.147	0.03	0.55
	dxyz		11	0.246	±0.146	0.13	0.56	0.266	±0.137	0.14	0.55
	dxyz		14	0.144	±0.120	0.04	0.43	0.143	±0.146	0.03	0.49
	x		total	−0.079	±0.085	−0.21	0.06	−0.080	±0.100	−0.23	0.07
	y		total	0.057	±0.040	−0.02	0.15	0.061	±0.041	−0.02	0.15
	z		total	0.089	±0.175	−0.09	0.52	0.113	±0.169	−0.06	0.49
	dxyz	100 µm	total	0.129	±0.067	0.03	0.22	0.151	±0.061	0.06	0.23
	dxyz		11	0.189	±0.022	0.16	0.22	0.202	±0.025	0.17	0.23
	dxyz		14	0.068	±0.036	0.03	0.13	0.099	±0.043	0.06	0.18
	x		total	−0.075	±0.093	−0.21	0.04	−0.057	±0.010	−0.21	0.10
	y		total	0.067	±0.039	0.0	0.13	0.070	±0.046	−0.02	0.15
	z		total	0.011	±0.027	−0.03	0.05	0.044	±0.041	−0.03	0.13
SHERA	dxyz	50 µm	total	0.091	±0.043	0.04	0.17	0.133	±0.052	0.04	0.24
	dxyz		11	0.098	±0.052	0.04	0.17	0.128	±0.048	0.04	0.15
	dxyz		14	0.083	±0.034	0.05	0.19	0.138	±0.060	0.04	0.24
	x		total	−0.048	±0.046	−0.13	0.03	−0.034	±0.083	−0.15	0.09
	y		total	0.009	±0.061	−0.10	0.11	0.026	±0.036	−0.02	0.11
	z		total	0.014	±0.039	−0.04	0.11	0.079	±0.062	−0.02	0.23
	dxyz	100 µm	total	0.183	±0.070	0.09	0.40	0.205	±0.069	0.07	0.35
	dxyz		11	0.230	±0.070	0.17	0.40	0.261	±0.047	0.20	0.35
	dxyz		14	0.136	±0.034	0.09	0.19	0.149	±0.036	0.07	0.20
	x		total	−0.097	±0.069	−0.19	−0.01	−0.094	±0.095	−0.23	0.02
	y		total	0.018	±0.048	−0.06	0.09	0.025	±0.034	−0.06	0.09
	z		total	0.132	±0.064	0.05	0.35	0.156	±0.048	0.06	0.27
FORM	dxyz	50 µm	total	0.290	±0.115	0.07	0.49	0.294	±0.114	0.12	0.48
	dxyz		11	0.271	±0.121	0.07	0.46	0.234	±0.092	0.12	0.48
	dxyz		14	0.308	±0.118	0.11	0.49	0.353	±0.111	0.19	0.48
	x		total	−0.126	±0.053	−0.24	−0.05	−0.078	±0.049	−0.19	0.02
	y		total	−0.007	±0.070	−0.14	0.13	−0.005	±0.071	−0.15	0.11
	z		total	0.242	±0.122	0.01	0.44	0.261	±0.127	0.04	0.44
	dxyz	100 µm	total	0.233	±0.090	0.10	0.47	0.262	±0.085	0.11	0.45
	dxyz		11	0.199	±0.086	0.10	0.41	0.222	±0.099	0.11	0.45
	dxyz		14	0.266	±0.090	0.14	0.47	0.302	±0.052	0.25	0.40
	x		total	−0.065	±0.078	−0.24	0.08	−0.032	±0.046	−0.12	0.08
	y		total	0.028	±0.073	−0.15	0.15	−0.024	±0.100	−0.20	0.13
	z		total	0.187	±0.100	0.04	0.42	0.225	±0.107	0.05	0.44

**Table 3:** Calculated medians and interquartile range (IQR) of the Kruskal–Wallis test for each of the tested groups before and after sterilization.

	Pre-Sterilization		Post-Sterilization	
Machine Type	Median [mm]	IQR [mm]	Median [mm]	IQR [mm]
MCX5	0.365	0.075	0.375	0.072
ASIGA	0.145	0.105	0.170	0.128
SHERA	0.135	0.093	0.155	0.065
FORM	0.240	0.140	0.260	0.163

**Table 4:** Results of the post hoc analysis between the groups before and after sterilization; level of significance: *p* ≤ 0.0125.

		*p*-Values	
Reference	Comparison	Pre-Sterilization	Post-Sterilization
MCX5	FORM	=0.022	=0.007
	ASIGA	<0.001	<0.001
	SHERA	<0.001	<0.001
FORM	ASIGA	<0.001	<0.001
	SHERA	<0.001	<0.001
ASIGA	SHERA	=0.404	=0.774

## Data Availability

All data obtained in this study are included in the article. Further requests can be addressed to the corresponding author.

## Abbreviations

The following abbreviations are used in this manuscript:

CAD	Computer-aided design
CAM	Computer-aided manufacturing
CBCT	Cone beam computed tomography
SLA	Stereolithography apparatus
DLP	Digital light processing
DMD	Direct micromirror device
DICOM	Digital imaging and communications in medicine
CEREC	Ceramic reconstruction
STL	Standard tessellation language
s-CAIS	Static computer-aided implant surgery
CNC	Computer numeric control
IQR	Interquartile range
